# Supplementary material for: Design of a Microbial Remediation Inoculation Program for Petroleum Hydrocarbon Contaminated Sites Based on Degradation Pathways
Source: Int J Environ Res Public Health. 2021 Aug 20;18(16):8794. doi: 10.3390/ijerph18168794 (PMC8395025; doi:10.3390/ijerph18168794)
Supplement: Supplementary file 1 [file ijerph-18-08794-s001.zip › ijerph-1298508-supplementary.pdf]

```java
package com.fish.tool;

import java.util.ArrayList;
import java.util.Arrays;
import java.util.List;

/**
 * @author lishiyao
 * @date 2021/5/26
 * @description
 */
public class Test {

	public static void main(String[] args) {
		List<List<Integer>> combine = combine(18, 4);
		List<Integer> group1 = Arrays.asList(1, 2, 3, 4, 5);
		List<Integer> group2 = Arrays.asList(6, 7, 8, 9, 10);
		List<Integer> group3 = Arrays.asList(11, 12, 13, 14, 15);
		List<Integer> group4 = Arrays.asList(16, 17, 18);
		for (List<Integer> group : combine) {
			boolean flog = false;
			boolean finalResult = false;
			finalResult = checkGroup(group1, group, flog, finalResult);
			finalResult = checkGroup(group2, group, flog, finalResult);
			finalResult = checkGroup(group3, group, flog, finalResult);
			finalResult = checkGroup(group4, group, flog, finalResult);
			if (finalResult == false) {
				System.out.println(group);
			}
			finalResult = false;
		}
	}

	private static boolean checkGroup(List<Integer> group1, List<Integer> group, boolean
flog, boolean finalResult) {
		for (Integer one : group1) {
			if (finalResult) {
				break;
			}
			if (group.contains(one) && flog) {
				finalResult = true;
				break;
			}
			if (group.contains(one)) {
```

```
                flog = true;
            }
        }
        return finalResult;
    }

    public static List<List<Integer>> combine(int n, int k) {
        List<List<Integer>> res = new ArrayList<List<Integer>>();
        List<Integer> list = new ArrayList<Integer>();
        //dfs
        dfs(1, n, k, list, res);
        return res;
    }

    public static void dfs(int start, int n, int k, List<Integer> list, List<List<Integer>> res) {
        if (list.size() == k) {
            res.add(new ArrayList<Integer>(list));
            return;
        }
        if (start > n) {
            return;
        }
        for (int i = start; i <= n; i++) {
            list.add(i);
            dfs(i + 1, n, k, list, res);
            list.remove(list.size() - 1);
        }
        return;
    }
}
```
